# Announcements

**Published:** 2015-05-01

## Global Road Safety Week — May 4–10, 2015

The United Nations Road Safety Collaboration has declared May 4–10, 2015, as the third United Nations Global Road Safety Week (GRSW). With the theme #SaveKidsLives, this year’s GRSW is dedicated to children, focusing on their safety on the world’s roads, actions that better ensure this, and the promotion for inclusion of safe and sustainable transport in the U.N.’s post-2015 development agenda (1).

The #SaveKidsLives campaign was launched in November 2014. With input from children and road safety experts around the world, the United Nations Road Safety Collaboration created the Child Declaration for Road Safety, calling on world leaders to include road safety in the global development agenda and outlining what can be done to increase road safety. The campaign invites all persons to read the declaration, sign it, and deliver it to those in charge of road safety in their countries and communities during GRSW (2). WHO also released a report, Ten Strategies for Keeping Children Safe on the Road, to guide stakeholders in their prevention and promotion efforts (3).

During this year’s GRSW, a regional congress focused on child road safety in the Americas will be held in Costa Rica. The congress will bring together key leaders from government agencies, non-governmental organizations, and the private sector to build capacity, share best practices, and create a consensus document on next steps for collaboration in the region.*

The U.N.’s GRSW is part of the organization’s larger Decade of Action for Road Safety 2011–2020 activities, aimed at saving five million lives on the road by the year 2020 (4). Connect with CDC’s Injury Center on Twitter at @CDCInjury to get safety tips leading up to and during Global Road Safety Week. Look for opportunities to get involved with groups in your community, such as the Safe Kids Coalition in the United States.†

Additional information about GRSW and the United Nations Decade of Action for Road Safety, as well as ideas on how to get involved in promoting road safety for children is available from the World Health Organization.§

The CDC is a partner in the global and U.S. efforts to improve road safety and prevent traffic injuries. More information and resources are available on CDC’s website.§

## Footnotes

* Additional information available at http://www.childroadsafetycongress.org.

† Additional information available at http://www.safekids.org/coalitions.

§ Additional information available at http://www.cdc.gov/features/globalroadsafety and http://www.cdc.gov/motorvehiclesafety.
